# Supplementary material for: Survey of Ticks and Tick-Borne Rickettsial and Protozoan Pathogens in Eswatini
Source: Pathogens. 2021 Aug 17;10(8):1043. doi: 10.3390/pathogens10081043 (PMC8401679; doi:10.3390/pathogens10081043)
Supplement: Supplementary file 1 [file pathogens-10-01043-s001.zip › pathogens-1317264-supplementary.pdf]

Supplementary Materials

Table S1. Sampling effort and ticks collected in Eswatini during June and July 2018 survey by site. The number of *Rhipicephalus* nymphs extracted and tested for tick-borne pathogens are represented in parentheses. .

				Amblyomma hebraeum			*Haemaphysalis elliptica*	*Rhipicephalus appendiculatus*	*Rhipicephalus evertsi*	*Rhipicephalus muehlensi*	*Rhipicephalus maculatus*	*Rhipicephalus simus*	*Rhipicephalus* sp.		
Type	Location	Site	distance sampled (m)	adult	nymph	larvae	adult	adult	adult	adult	adult	adult	nymphs (extracted nymphs)	larvae w/ festoons	larvae w/o festoons
wildlife conservation area	Hlane	HH1	1200	1	0	45	1	3	1	8	1	10	42 (20)	586	11
		HH2	1200	0	0	0	0	3	0	2	5	8	107 (20)	3665	5
		HM1	1200	1	2	15	1	1	0	0	0	84	1 (1)	120	120
		HH3	1200	3	3	0	7	18	0	1	5	47	21 (18)	0	0
		LL1	1200	0	0	3992	0	2	0	1	0	42	69 (20)	642	313
		ML1	1200	0	0	2	0	6	1	1	3	12	1 (1)	74	4
		MM1	1200	1	0	0	2	2	0	0	0	35	0 (0)	37	26
	Mbluluzi	HL1	1200	0	4	1484	0	33	0	2	5	5	18 (18)	13941	206
		HL2	1200	0	0	1246	1	18	0	1	0	5	399 (20)	395	0
		HM2	1200	0	3	1418	0	0	0	0	0	1	159 (20)	2051	41
	Mlawula	LL2	1200	0	1	5	1	0	0	1	5	4	23 (19)	909	25
		ML2	1200	0	1	4	45	1	0	1	1	18	69 (20)	770	20
		MLA	600	0	1	284	0	0	0	4	11	1	220 (60)	670	830
	Dombeya	DO	1000	0	2	245	6	23	1	2	0	0	351 (60)	2283	51
	Mhlosinga	MH	1000	0	1	7	11	6	0	0	0	7	1 (1)	400	6
	Nisela	NI	1200	0	0	56	10	15	0	0	0	4	0 (0)	52	264

mixed cattle and game ranch	IYSIS	LH1	1200	0	0	973	0	0	0	0	0	0	6 (6)	461	4
		LH2	1200	0	5	0	0	0	0	0	0	0	69 (20)	1793	262
		LM1	1200	0	0	342	2	0	0	0	0	0	54 (20)	898	1967
		LM2	1200	0	2	0	2	0	0	0	2	1	58 (20)	609	470
	Bushlands Ranch	BU2	300	0	1	8	4	0	0	0	0	0	0 (0)	582	0
cattle ranch	Nkalashane Ranch	NK	1200	0	0	818	43	0	0	0	0	13	0 (0)	0	54
	Ndukuyamangendla Ranch	RIC	1200	0	1	508	9	0	0	0	0	18	1 (1)	0	0
communal	Lomahasha	LO1	300	0	1	528	2	1	0	0	0	0	0 (0)	8	886
	Maphiveni	MA1	400	0	0	0	32	2	0	0	0	1	0 (0)	0	0
	Ndzevane	SE	800	0	0	0	1	0	0	0	0	0	0 (0)	252	7
	Sitsatsaweni	SI	600	0	0	1019	33	0	0	0	0	2	3 (3)	10	214

Table S2. Sampling effort and ticks collected in Eswatini during December 2018 and June and July 2019 surveys by site in wildlife conservation areas. The number of *Rhipicephalus* nymphs extracted and tested for tick-borne pathogens are represented in parentheses. .

				Amblyomma hebraeum			*Haemaphysalis elliptica*	*Rhipicephalus appendiculatus*	*Rhipicephalus evertsi*	*Rhipicephalus muehlensi*	*Rhipicephalus maculatus*	*Rhipicephalus simus*	*Rhipicephalus* sp.		
Session	Location	Site	distance sampled (m)	adult	nymph	larvae	adult	adult	adult	adult	adult	adult	nymphs (extracted nymphs)	larvae w/ festoons	larvae w/o festoons
December 2018	Hlane	HH1	800	0	2	0	0	2	0	1	17	22	3 (3)	0	179
		HM1	800	0	1	137	3	1	0	1	0	35	5 (5)	8	1076
		HH3	800	4	0	63	0	0	0	0	4	15	11 (11)	87	1853
		LL1	800	0	0	914	0	1	0	0	0	22	19 (19)	172	2278
	Mbluluzi	HL1	800	0	4	4575	0	2	0	4	9	0	99 (20)	0	226
		HL2	800	0	1	1610	0	0	0	1	3	1	136 (20)	226	575
		HM2	800	0	0	349	0	0	0	0	6	1	145 (20)	20	101
	IYSIS	LH2	800	0	0	350	0	0	1	0	1	0	3 (3)	42	511
		LM2	800	0	0	18	0	0	0	0	6	0	10 (10)	306	400
	Mlawula	LL2	800	0	0	0	0	0	0	3	7	3	102 (20)	97	570
		ML2	800	1	2	8	0	0	0	3	1	26	165 (20)	895	588
June and July 2019	Hlane	HH1	400	0	0	0	0	0	0	0	0	1	9 (9)	704	57
		HM1	400	0	0	27	0	1	0	0	0	21	0 (0)	28	1
		HH3	400	0	0	4	1	0	1	1	0	3	6 (6)	350	72
		LL1	400	0	0	649	0	0	0	1	0	12	24 (20)	555	98
	Mbluluzi	HL1	400	0	0	196	2	1	1	0	0	2	30 (20)	1864	432
		HL2	400	0	0	168	0	1	0	0	0	0	36 (20)	1009	68

		HM2	400	1	0	59	0	2	0	0	0	4	114 (20)	2758	39
	IYSIS	LH2	400	0	0	32	0	0	0	0	0	0	17 (17)	202	464
		LM2	400	0	0	23	0	1	0	0	0	0	33 (20)	316	553
	Mlawula	LL2	400	0	0	54	0	0	0	0	1	2	3 (3)	1671	77
		ML2	400	0	0	129	0	0	0	0	0	6	15 (15)	483	73

Table S3. Density of questing ticks in protected versus unprotected sites during June and July 2018. Bold represents statistical significance.

Tick Species and Life Stage	Protected area sample mean density (per 100m)	Unprotected area sample mean density (per 100m)	t-test; df = 25
Amblyomma hebraeum larvae	41.6	76.0	t = -1.1; p = 0.3
Rhipicephalus (*Boophilus*) larvae	21.7	56.0	t = -0.2; p = 0.8
Rhipicephalus larvae	134.5	6.0	**t = 4.0; p < 0.001**
Rhipicephalus nymphs	7.7	0.1	**t = 2.6; p = 0.014**
Rhipicephalus appendiculatus adult	0.89	0.12	t = 1.2; p = -0.25
Rhipicephalus evertsi adult	0.02	0	NA
Rhipicephalus maculatus adult	0.22	0	NA
Rhipicephalus muehlensi adult	0.17	0	NA
Rhipicephalus simus adult	1.14	0.53	t = 0.7; p = 0.5
Haemaphysalis elliptica adult	0.43	3.1	**t = -3.6; p = 0.001**

Table S4. Mean density of questing ticks at resampled sites. Paired t-tests with 95% confidence levels. Bold represents statistical significance.

Tick Species and Life Stage	Repeated sites: 2018 (per 100m)	Repeated sites: 2019 (per 100m)	t-test; df = 10	Repeated sites: Winter 2018 (per 100m)	Repeated sites: Summer 2018 (per 100m)	t-test; df = 10
Amblyomma hebraeum larvae	62.2	30.5	t = -0.9; p = 0.4	62.2	91.2	t = -1.5; p = 0.2
Rhipicephalus (*Boophilus*) larvae	11.1	44.0	**t = -3.1; p = 0.01**	11.1	95.0	**t = -4.9; p < 0.001**
Rhipicephalus larvae	165.3	225.9	t = -2.1; p = 0.07	165.3	21.1	**t = 2.3; p = 0.05**
Rhipicephalus nymphs	6.98	6.55	t = 0.1, p = 0.9	6.98	8.09	t = -0.06, p = 0.9
Rhipicephalus appendiculatus adult	0.57	0.14	t = 1.49; p = 0.2	0.57	0.07	t = 2.0; p = 0.07
Rhipicephalus evertsi adult	0.02	0.05	t = -0.8; p = 0.4	0.02	0.01	t = 0.2; p = 0.8
Rhipicephalus maculatus adult	0.14	0.02	**t = 2.6; p = 0.03**	0.14	0.61	**t = -3.1; p = 0.01**
Rhipicephalus muehlensi adult	0.11	0.05	t = 1.1; p = 0.3	0.11	0.15	t = -0.53; p = 0.6
Rhipicephalus simus adult	1.64	1.16	t = 1.1; p = 0.3	1.64	1.42	t = 0.37; p = 0.7
Haemaphysalis elliptica adult	0.44	0.07	t = 1.2; p = 0.3	0.44	0.03	t = 1.4; p = 0.2

Table S5. The percent sequence identity of identified bacterial and protozoan species in this study to reference sequences obtained from Genbank. The percent sequence identity describes how similar a sequence from this study is to a reference sequence and was calculated for each gene from pathogen species by dividing the number of matching nucleotides between the sequence obtained in this study and the reference sequence by the total number of nucleotides.

Pathogen Species/Genotypes	Gene	Reference Genbank Accession Number	Sequence Identity
Rickettsia africae	*ompA*	CP001612	99.3-100%
	ompB	CP001612	100%
	gltA	CP001612	100%
Candidatus *Rickettsia barbariae*	*ompA*	EU272186	99.3%
	ompB	EU272187	100%
	gltA	EU272185	99.3%
Rickettsia conorii	*ompA*	AE006914	99.6-100%
	ompB	AE006914	100%
	gltA	AE006914	100%
Rickettsia massiliae	*ompA*	CP000683	100%
	ompB	CP000683	100%
	gltA	CP000683	99.5-100%
Rickettsia sp. A	*ompA*	CP019435, *R. raoulti*	97.6%
	ompB	CP019435, *R. raoulti*	98.5%
	gltA	CP019435, *R. raoulti*	99.6%
Rickettsia sp. B	*ompA*	KT835137, *Rickettsia* sp. SSSE	100%
	ompB	CP013133, *Rickettsia rhipicephali*	99.2%
	gltA	CP013133, *R. rhipicephali*	99.8%
Rickettsia sp. C	*ompA*	NC_017065, *R. slovaca*	92.2%
	ompB	NC_017065, *R. slovaca*	94.5-96.8%
	gltA	NC_017065, *R. slovaca*	99.4%
Ehrlichia ruminantium	*groEL*	NC_005295	98.3%
Ehrlichia minasensis	*16S*	MH500005	99.7-100%
	groEL	JX629806	98.6%
Babesia caballi from *R. simus*	*18S*	EU642514	98.6%
		KX375825	98.8%
Babesia caballi from *R. appendiculatus*	*18S*	EU642514	97.1%
		KX375825	97.3%
Babesia sp.	*18S*	HQ437690, *Babesia* sp.	99.0%
Hepatozoon sp.	*18S*	KF939627, *Hepatozoon* sp.	98.8%
		MN723845, *Hepatozoon ophisauri*	98.8%
		MN791089, *Hepatozoon canis*	92.2%
Theileria equi	*18S*	EU888902	99.0%
Theileria ovis	*18S*	AY260172	99.2%
Theileria taurotragi	*18S*	MH393312 and MK131255	100%

Theileria velifera	*18S*	MN595045	99.7%
Theileria sp. (sable)	*18S*	GU733378	99.2%
Theileria sp. (giraffe)	*18S*	FJ213582	100%
Theileria sp. (waterbuck)	*18S*	KF597072	100%
Theileria sp. Tragelaphini B	*18S*	MK131247	98.7%
Theileria sp. Tragelaphini D	*18S*	MK131250	98.7-100%
Theileria sp. Tragelaphini E	*18S*	MK131251	99.3-99.7%
Theileria sp. Aepycerotini	*18S*	MK131245	100%
Theileria sp. (impala)	*18S*	MK131253	100%
Theileria sp. (rodent)	*18S*	MT269267	99.5%
		MK484070	98.8%
Theileria sp. A	*18S*	EU053199, *Theileria* sp. (dog)	96.9%
		GU733378, *Theileria* sp. (sable)	95.6%
Theileria sp. B	*18S*	MK131255, *Theileria taurotragi*	96.9%

Table S6. Tick infection prevalence (no. infected/no. tested), with 95% confidence intervals for prevalence estimates in brackets, considering all adult ticks and the subset of nymphal ticks tested for tick-borne pathogens by site and sampling session.

		Tick Infection Prevalence		
Location	Site	Winter 2018	Summer 2018	Winter 2019
Hlane	HH1	4/45; 8.9% [2.5-21.2%]	4/47; 8.5% [2.4-20.4%]	1/10; 10%[0.3-44.5%]
	HH2	11/38; 28.9% [15.4-45.9%]	-	-
	HM1	23/90; 25.6% [16.9-35.8%]	6/45; 13.3% [5.1-26.8%]	3/22; 13.6%[2.9-34.9%]
	HH3	25/103; 24.3% [16.4-33.7%]	6/34; 17.6% [6.8-34.5%]	3/12; 25%[5.5-57.2%]
	LL1	15/65; 23.1% [13.5-35.2%]	5/42; 11.9% [4-25.6%]	7/33; 21.2%[9-38.9%]
	ML1	6/24; 25% [9.8-46.7%]	-	-
	MM1	9/40; 22.5% [10.8-38.5%]	-	-
Mbluluzi	HL1	21/67; 31.3% [20.6-43.8%]	9/39; 23.1% [11.1-39.3%]	4/26; 15.4%[4.4-34.9%]
	HL2	10/45; 22.2% [11.2-37.1%]	4/26; 15.4% [4.4-34.9%]	6/21; 28.6%[11.3-52.2%]
	HM2	5/24; 20.8% [7.1-42.2%]	0/28; 0% [0-12.3%]	5/27; 18.5%[6.3-38.1%]
Mlawula	LL2	7/31; 22.6% [9.6-41.1%]	6/33; 18.2% [7-35.5%]	1/6; 16.7%[0.4-64.1%]
	ML2	16/87; 18.4% [10.9-28.1%]	5/53; 9.4% [3.1-20.7%]	2/21; 9.5%[1.2-30.4%]
	MLA	16/77; 20.8% [12.4-31.5%]	-	-
Dombeya Nature Estate	DO	24/94; 25.5% [17.1-35.6%]	-	-
Mhlosinga Nature Reserve	MH	5/26; 19.2% [6.6-39.4%]	-	-
Nisela Game Reserve	NI	4/29; 13.8% [3.9-31.7%]	-	-
IYSIS	LH1	0/6; 0% [0-45.9%]	-	-
	LH2	5/25; 20% [6.8-40.7%]	0/5; 0% [0-52.2%]	3/17; 17.6% [3.8-43.4%]
	LM1	2/22; 9.1% [1.1-29.2%]	-	-
	LM2	3/27; 11.1% [2.4-29.2%]	0/16; 0% [0-20.6%]	3/21; 14.3% [3-36.3%]
Bushlands Ranch	BU2	3/5; 60% [14.7-94.7%]	-	-
Nkalashane Ranch	NK	23/56; 41.1% [28.1-55%]	-	-

Ndukuyamangendla Ranch	RIC	6/29; 20.7% [8-39.7%]	-	-
Lomahasha	LO1	0/4; 0% [0-60.2%]	-	-
Maphiveni	MA1	17/35; 48.6% [31.4-66%]	-	-
Ndzevane	SE	0/1; 0% [0-97.5%]	-	-
Sitsatsaweni	SI	5/38; 13.2% [4.4-28.1%]	-	-

Table S7. The number and species identity of ticks with coinfections of pathogen species belonging to *Anaplasma, Ehrlichia, Rickettsia,* and *Theileria* observed among 1716 ticks.

	Anaplasma sp.	Ehrlichia sp.	Theileria velifera	Theileria sp. waterbuck	Theileria ovis	Theileria sp. Tragelaphini B	Theileria sp. Tragelaphini D	Theileria taurotragi	Theileria sp. Aepycerotini	Theileria sp. Tragelaphini E
Rickettsia barbariae	1 R. simus	-	-	-	-	-	-	-	-	-
Rickettsia conorii	5 H. elliptica	-	-	-	-	-	-	-	-	-
Rickettsia sp. B	1 R. simus	-	-	-	-	-	-	-	-	-
Rickettsia massiliae	-	1 R. simus	-	-	-	-	-	-	-	-
Rickettsia africae	-	-	3 A. hebraeum	2 A. hebraeum	-	-	-	-	-	-
Anaplasma sp.	-	-	-	-	1 R. appendiculatus	-	-	-	-	-
Ehrlichia sp.	-	-	-	-	-	1 R. muehlensi	2 R. appendiculatus	1 R. appendiculatus	2 R. appendiculatus	-
Ehrlichia minasensis	-	-	-	-	-	-	-	-	-	1 R. simus

Table S8. Conventional PCR primers and reaction conditions used in this study. All conventional PCR assays were performed in 25 μL reactions, using 5 μL of 5x GoTaq® Reaction Buffer and 200 μM of each dNTP. Cycling conditions were as follows: 95°C for 3 min; specified number cycles of 95°C for 30 seconds (except for Tick ITS2 for 60 seconds and Tick 12S for 15 seconds), specified annealing conditions, and specific elongation; followed by 72 °C for 7 min and hold at 4 °C.

**Target Organism**	**Target Gene**	**Primer Name**	**Primer Orientation**	**5′-3′ sequence**	**Ref**	**Primer Conc (nM)**	**$MgCl_2$ Conc (mM)**	**GoTaq® DNA polymerase (U)**	**Cycle s**	**Annealing (temp [°C], time [s])**	**Elongation (temp [°C], time [s])**
Tick	ITS2	3SAF	Forward	CTAAGCGGTGGATCACTCGG	[1]	500	2.25	0.2	35	55, 60	72, 120
		ITS2R	Reverse	ATATGCTTAAATTCAGCGGG	[2]	500					
Tick	12S	T1B	Forward	AAACTAGGATTAGATACCC	[3]	100	3.25	0.5	5	51, 30	68, 30
		T2A	Reverse	AATGAGAGCGACGGGCGATGT		100			25	53, 30	70, 30
Tick	CO1	LEP-F1	Forward	ATTCAACCAATCATAAAGATATTGG	[4]	500	2.5	1.0	35	54, 30	72, 60
		LEP-R1	Reverse	TAAACTTCTGGATGTCCAAAAAATCA		500					
Tick	CO1	Chel-CO1-F1	Forward	TACTCTACTAATCATAAAGACATTGG	[5]	500	2.5	1.0	5	45, 45	72, 60
		Chel-CO1-R1	Reverse	CCTCCTCCTGAAGGGTCAAAAAATGA		500			35	50, 45	72, 60
Rickettsia.	ompA	Rr190.70p	Forward	ATGGCGAATATTTCTCCAAAA	[6]	600	2.25	0.625	40	54.8, 45	72, 30
		90.701	Reverse	GTTCCGTTAATGGCAGCATCT	[7]	600					
Rickettsia	ompB	120-M59	Forward	CCGCAGGGTTGGTAACTGC	[8]	300	2.0	1.25	40	50, 30	72, 60
		120-807	Reverse	CCTTTTAGATTACCGCCTAA		300					
Rickettsia.	gltA	CS409d	Forward	CCTATGGCTATTATGCTTGC	[9]	200	2.25	1.25	40	53, 30	72, 55
		Rp1258n	Reverse	ATTGCAAAAAGTACAGTGAACA		200					
Anaplasmataceae	16S	Ehr-16S-D	Forward	GGTACCYACAGAAGAAGTCC	[10]	200	2.25	1.25	40	54, 60	72, 60
		Ehr-16S-R	Reverse	TAGCACTCATCGTTTACAGC		200					
Anaplasma.	rpoB	Ana-rpoBF	Forward	GCTGTTCCTAGGCTYTCTTACGCGA	[11]	500	2.25	1.25	40	55, 60	72, 60
		Ana-rpoBR	Reverse	AATCRAGCCAVGAGCCCCTRTAWGG		500					
Ehrlichia	groEL	Ehr-groEL-F	Forward	GTTGAAAARACTGATGGTATGCA	[11]	500	2.25	1.25	40	50, 60	72, 60
		Ehr-groEL-R	Reverse	ACACGRTCTTTACGYTCYTTAAC		500					
	18S	ILO-9029	Forward 1	CGGTAATTCCAGCTCCAATAGCGT	[12]	200	1.5	1.0	30	53, 30	72, 60
		ILO-9030	Reverse 1	TTTCTCTCAAGGTGCTGAAGGAGT		200					

Apicomplexa (*Babesia, Theileria, Hepatozoon*)	(nested cPCR)	MWG4-70	Forward 2	AGCTCGTAGTTGAATTTCTGCTGC		200	1.5	1.0	30	55,30	72, 60
		ILO-7782	Reverse 2	AACTGACGACCTCCAATCTCTAGTC		200					

Table S9. Real-time PCR primers, probes, and reaction conditions used in this study.

Target Organism	Target Gene	Primer Name	Primer Orientation	5′-3′ sequence	Ref	Primer/Probe Concentration (nM)	Initial Denature	Denaturation, annealing, and extension
Anaplasmataceae	23S	Tt-Ana-F	Forward	TGACAGCGTACCTTTTGCAT	[13]	200	95 °C for 10 min	40 cycles of 95 °C for 15 seconds and 60 °C for 1 min
		Tt-Ana-R	Reverse	GTAACAGGTTCGGTCCTCCA		200		
		Tt-Ana-S	Probe	6-FAM™-GGATTAGAC/ZEN/CCGAAACCAAG-Iowa Black®		250		
Rickettsia	17-kd	R17K135F	Forward	ATGAATAAACAAGGKACNGGHACAC	[14]	800	95 °C for 15 min	40 cycles of 95 °C for 10 seconds, 60°C for 10 sec, and 72 °C for 30 sec
		R17K249R	Reverse	AAGTAATGCRCCTACACCTACTC		800		
		R17KbC	Probe	6-FAM-TTGGTTCTCAATTCGGTAAGGGTAAAGG – Black Hole Quencher®		100		

References

1. Barker, S.C. Distinguishing species and populations of Rhipicephaline ticks with its 2 ribosomal RNA. *Journal of Parasitology* **1998**, *84*, 887–892, doi:10.2307/3284614.
2. Domanico, M.J.; Phillips, R.B.; Oakley, T.H. Phylogenetic analysis of Pacific salmon (genus *Oncorhynchus*) using nuclear and mitochondrial DNA sequences. *Canadian Journal of Fisheries and Aquatic Sciences* **1997**, *54*, 1865–1872, doi:10.1139/cjfas-54-8-1865.
3. Beati, L.; Keirans, J.E. Analysis of the systematic relationships among ticks of the genera *Rhipicephalus* and *Boophilus* (Acari : Ixodidae) based on mitochondrial 12S ribosomal DNA gene sequences and morphological characters. *Journal of Parasitology* **2001**, *87*, 32–48, doi:10.2307/3285173.
4. Hebert, P.D.N.; Penton, E.H.; Burns, J.M.; Janzen, D.H.; Hallwachs, W. Ten species in one: DNA barcoding reveals cryptic species in the neotropical skipper butterfly *Astraptes fulgerator*. *Proceedings of the National Academy of Sciences of the United States of America* **2004**, *101*, 14812–14817, doi:10.1073/pnas.0406166101.
5. Barrett, R.D.H.; Hebert, P.D.N. Identifying spiders through DNA barcodes. *Canadian Journal of Zoology* **2005**, *83*, 481–491, doi:10.1139/z05-024.
6. Regnery, R.L.; Spruill, C.L.; Plikaytis, B.D. Genotypic identification of Rickettsiae and estimation of intraspecies sequence divergence for portions of two rickettsial genes. *Journal of Bacteriology* **1991**, *173*, 1576–1589, doi:10.1128/jb.173.5.1576-1589.1991.
7. Roux, V.; Fournier, P.E.; Raoult, D. Differentiation of spotted fever group rickettsiae by sequencing and analysis of restriction fragment length polymorphism of PCR-amplified DNA of the gene encoding the protein rOmpA. *Journal of Clinical Microbiology* **1996**, *34*, 2058–2065, doi:10.1128/jcm.34.9.2058-2065.1996.
8. Roux, V.; Raoult, D. Phylogenetic analysis of members of the genus *Rickettsia* using the gene encoding the outer-membrane protein rOmpB (ompB). *International Journal of Systematic and Evolutionary Microbiology* **2000**, *50*, 1449–1455, doi:10.1099/00207713-50-4-1449.
9. Roux, V.; Rydkina, E.; Eremeeva, M.; Raoult, D. Citrate synthase gene comparison, a new tool for phylogenetic analysis, and its application for the Rickettsiae. *International Journal of Systematic Bacteriology* **1997**, *47*, 252–261, doi:10.1099/00207713-47-2-252.

10. Parola, P.; Cornet, J.P.; Sanogo, Y.O.; Miller, R.S.; Thien, H.V.; Gonzalez, J.P.; Raoult, D.; Telford, S.R.; Wongsrichanalai, C. Detection of *Ehrlichia* spp., *Anaplasma* spp., *Rickettsia* spp., and other eubacteria in ticks from the Thai-Myanmar border and Vietnam. *Journal of Clinical Microbiology* **2003**, *41*, 1600–1608, doi:10.1128/jcm.41.4.1600-1608.2003.
11. Dahmani, M.; Davoust, B.; Rousseau, F.; Raoult, D.; Fenollar, F.; Mediannikov, O. Natural Anaplasmataceae infection in *Rhipicephalus bursa* ticks collected from sheep in the French Basque Country. *Ticks and Tick-Borne Diseases* **2017**, *8*, 18–24, doi:10.1016/j.ttbdis.2016.09.009.
12. Hawkins, E.; Kock, R.; McKeever, D.; Gakuya, F.; Musyoki, C.; Chege, S.M.; Mutinda, M.; Kariuki, E.; Davidson, Z.; Low, B.; et al. Prevalence of *Theileria equi* and *Babesia caballi* as well as the identification of associated ticks in sympatric Grevy's zebras (*Equus grevyi*) and donkeys (*Equus africans asinus*) in northern Kenya. *Journal of Wildlife Diseases* **2015**, *51*, 137–147, doi:10.7589/2013-11-316.
13. Dahmani, M.; Davoust, B.; Benterki, M.S.; Fenollar, F.; Raoult, D.; Mediannikov, O. Development of a new PCR-based assay to detect Anaplasmataceae and the first report of *Anaplasma phagocytophilum* and *Anaplasma platys* in cattle from Algeria. *Comparative Immunology Microbiology and Infectious Diseases* **2015**, *39*, 39–45, doi:10.1016/j.cimid.2015.02.002.
14. Loftis, A.D.; Reeves, W.K.; Szumlas, D.E.; Abbassy, M.M.; Helmy, I.M.; Mortarity, J.R.; Dasch, G.A. Surveillance of Egyptian fleas for agents of public health significance: *Anaplasma*, *Bartonella*, *Coxiella*, *Ehrlichia*, *Rickettsia*, and *Yersinia pestis*. *American Journal of Tropical Medicine and Hygiene* **2006**, *75*, 41–48, doi:10.4269/ajtmh.2006.75.41.
